# Electrospun Microfibers Modulate Intracellular Amino Acids in Liver Cells via Integrin β1

**DOI:** 10.3390/bioengineering8070088

**Published:** 2021-06-22

**Authors:** Tianjiao Huang, John A. Terrell, Jay H. Chung, Chengpeng Chen

**Affiliations:** 1 Laboratory of Obesity and Aging Research, Genetics and Development Biology Center, National Heart Lung and Blood Institute, National Institutes of Health, Bethesda, MD 20892, USA; tianjiao.huang@nih.gov (T.H.); chungj@nhlbi.nih.gov (J.H.C.); 2 The Department of Chemistry and Biochemistry, University of Maryland Baltimore County, Baltimore, MD 21250, USA; johnt5@umbc.edu

**Keywords:** electrospinning, microfibers, hepatocytes, amino acids, integrin

## Abstract

Although numerous recent studies have shown the importance of polymeric microfibrous extracellular matrices (ECMs) in maintaining cell behaviors and functions, the mechanistic nexus between ECMs and intracellular activities is largely unknown. Nevertheless, this knowledge will be critical in understanding and treating diseases with ECM remodeling. Therefore, we present our findings that ECM microstructures could regulate intracellular amino acid levels in liver cells mechanistically through integrin β1. Amino acids were studied because they are the fundamental blocks for protein synthesis and metabolism, two vital functions of liver cells. Two ECM conditions, flat and microfibrous, were prepared and studied. In addition to characterizing cell growth, albumin production, urea synthesis, and cytochrome p450 activity, we found that the microfibrous ECM generally upregulated the intracellular amino acid levels. Further explorations showed that cells on the flat substrate expressed more integrin β1 than cells on the microfibers. Moreover, after partially blocking integrin β1 in cells on the flat substrate, the intracellular amino acid levels were restored, strongly supporting integrin β1 as the linking mechanism. This is the first study to report that a non-biological polymer matrix could regulate intracellular amino acid patterns through integrin. The results will help with future therapy development for liver diseases with ECM changes (e.g., fibrosis).

## 1. Introduction

Although simple and straightforward, conventional cell cultures as 2D monolayers cannot replicate microenvironments in vivo, which may explain the high failure rates of translating in vitro cell−based results to clinical studies [1,2]. Recent evidence suggests that 3D scaffolding materials are essential to maintain the physiologically relevant functions of cell cultures in vitro [3]. To date, various materials and techniques have been developed as scaffolds to mimic extracellular matrices (ECMs); among these, the electrospinning method which can generate ultrathin fibers from a rich variety of materials such as synthetic (e.g., polystyrene) and natural (e.g., gelatin) polymers [4,5,6] has been widely adopted. Briefly, this technique uses a high voltage to induce the formation of a liquid jet (Taylor cone) at the tip of a blunt syringe needle, where a polymer solution is pumped out [7]. As the liquid jet stretches due to the electrostatic repulsions between the surface charges and the evaporated solvents, continuous fibers are formed assisted by the high viscosity of the polymer solution [8]. Electrospun fibers are considered as an ideal scaffold for cell cultures due to the simple setup and the capability to tune the fiber morphologies (diameter and porosity) [9].

To date, various cell types have been successfully cultured on electrospun scaffolds with enhanced activities and functions compared to 2D cultures [10,11]. Amongst these in vitro 3D cell cultures, hepatocytes have attracted substantial interest because of the high demand for reliable hepatic models in pharmaceutical and pathophysiological studies [12,13]. For instance, precise understanding of the hepatic metabolism and toxicity of a new drug candidate is critically important in drug development [14]. Moreover, 3D ECMs can modulate the fingerprint functions of hepatocytes, including albumin secretion, urea synthesis, cytochrome 450 (CYP450) activities, and metabolism of drugs [15,16,17]. Understanding the mechanism by which the 3D ECMs affect hepatocyte function can guide precise ECM designs for future 3D liver engineering. Moreover, many liver diseases involve ECM changes (e.g., cirrhosis) [18], and knowing the mechanistic link between ECM conditions and intracellular signaling will help find new targets for therapeutic development.

Here, we present our recent findings regarding the ECM–cell interaction mechanisms. We conducted amino acid metabolomics quantitation to demonstrate the role of a commonly used microfibrous ECM on hepatocytes. Amino acids are essential building blocks for the protein synthesis function of liver cells. Moreover, a recent study showed that amino acids could affect the overall metabolic activities, CYP450 activities, and cell maturation of hepatocytes [19]. We first found that the fibrous ECM generally increased the abundance of most of the amino acids investigated compared to those in samples from 2D flat substrates. Further mechanistic studies demonstrated that the microfiber-induced reduction of integrin expression altered the intracellular amino acid levels in hepatocytes. Overall, we report a new observation that 3D ECMs can modulate intracellular amino acids in hepatocytes. More importantly, the mechanistic role of integrins in bridging ECM materials and the intracellular variations was revealed. This new finding and insight lay a foundation for future investigations of cell–ECM interactions and hepatic modeling.

## 2. Experimental

### 2.1. ECM Preparation

Silk fibroin was extracted in−lab following the published protocol [20] from cocoons of Bombyx mori silkworms. The purified and lyophilized silk fibroin was dissolved in hexaisofluoropropanol (HFP; MilliporeSigma, MO, USA) at a final concentration of 6% (*w/v*). The solution was loaded in a 3 mL syringe (BD, Andover, MA, USA) coupled with a 26 Gauge stainless steel blunt needle (Fisher Scientific, Pittsburgh, PA, USA). A syringe pump (NewEra, Farmingdale, NY, USA) was applied to push the solution out at 1 mL/min. The needle was connected to a 25 kV power supply (Spellman, Valhalla, NY, USA) to electrospin fibers. A grounded stainless steel plate (6 × 6 in.) covered by a polystyrene sheet (150 µm thick) was placed 30 cm apart from the needle to collect the fibers. After 1 hr, a uniform layer of fibers was collected on the polystyrene film, which was laser cut (Full Spectrum Laser, 45W CO_2_, 17% vector current, Las Vegas, NV, USA) into 34.5 mm (fitting a 6−well plate) and 16.0 mm (fitting a 24−well plate) for subsequent cell cultures. Images of the fibers were taken under SEM (scanning electrical microscope, FEI, Hillsboro, OR, USA). The software ImageJ was then used to measure the fiber sizes. To prepare the control of 2D ECMs, blank polystyrene sheets were first cut into 34.5 and 16.0 mm circles, followed by pipetting 1 mL and 200 µL of the silk fibroin solution onto each disc, respectively. The solution was spread on the discs and dried in a fume hood after about 1 hr. After evaporation of the silk fibroin solution, discs were rinsed three times with MilliQ water and allowed to dry overnight in a sterile fume hood to remove any residual solvent present from electrospinning.

### 2.2. Cell Culturing

For the amino acid metabolomics experiments, the discs were cut to fit a 6−well plate to ensure sufficient cell numbers for the measurements. For the basic characterizations, including urea production, albumin secretion, CYP 450 activity, and cell proliferation, smaller discs fitting a 24−well plate were used. All the discs were thoroughly rinsed by 70% ethanol and air−dried in UV before cell culturing. Huh7 Cells (ATCC, Manassas, VA, USA; passage 3–6) were seeded onto discs modified to contain either silk fibroin fibers or a silk fibroin coating with no topography. For the 6−well plate format, about 100,000 cells (determined by a hemocytometer) were seeded in each well. For 24−well plates, 20,000 cells were added to each well. In a humid incubator at 37 °C and with 5% CO_2_, cells were cultured on the discs in well plates for one week in DMEM (Thermo Fisher Scientific, PA, USA; supplemented with 10% FBS and 1% Pen−strep) with media changed every 2–3 days. Once cells were confluent, media was decanted, and cells were washed with sterile PBS. The polystyrene discs with cells were then transferred to a new plate to avoid assay interference from cells which may have spread to the well plate surface and do not experience the desired ECM topography. Cells were then cultured in 1 mL of phenol red free DMEM (MilliporeSigma, St. Louis, MO, USA; supplemented with 10% fetal bovine serum and 1% Pen−strep) overnight.

### 2.3. Dead Cell Fluorescent Staining

The cell viability indicator ethidium homodimer−1 (Ethd−1) (Thermo Fisher Scientific, PA, USA) was used for dead cell staining following the manufacturer’s instructions. In brief, 2 mL PBS with 10 µM Ethd−1 and 300 nM DAPI (Thermo Fisher Scientific, Pittsburgh, PA, USA) was used to incubate cells on the fibers or on the flat surface for 20 min in a 6−well plate well. Then, cells on the substrates were rinsed for 3 times with PBS and examined with a fluorescent microscope (EVOS M5000, Thermo Fisher Scientific, Pittsburgh, PA, USA). The yellow–red emission channel (green excitation) was used to image Ethd−1−stained cells (dead cells). Next, UV excitation was turned on, followed by imaging with the blue channel for DAPI−stained cells (the total number of cells). Then, the percentages of dead and live cells were calculated.

### 2.4. BCA Assay

This was to determine cell numbers in each well for data normalization. Cells were rinsed three times with sterile water and then incubated for 30 min on ice in 600 µL of RIPA buffer solution. Samples were then further lysed using a sonic horn (1 s on, 4 s off for 1 min). An aliquot of 25 µL of each sample was transferred to a 96 well plate and combined with 200 µL of BCA solution (MilliporeSigma, St. Louis, MO, USA) to determine the protein concentration in each solution. Samples were incubated for 30 min and the absorbance read at λ = 562 nm. Based on a calibration curve previously developed by lysing known amounts of Huh7 cells and quantitating the amount of protein in the lysates, the number of cells was then determined per well to normalize the data from other assays.

### 2.5. Urea Assay

A total of 900 µL of media from each well was transferred to 1.7 mL Eppendorf tubes and lyophilized. The remaining solid was reconstituted in 100 µL methanol to dissolve urea while avoiding proteinaceous material. The methanol solution was collected after centrifuging, followed by air−drying at 4 °C and reconstitution in 100 µL of MilliQ water. In a 384−well plate, 80 µL of the reconstituted solution was added to a well and combined with 5 µL of both Urease solution (MilliporeSigma; 362 U/mL in PBS) and 0.12 mg/mL phenolphthalein. Samples were then incubated at room temperature for 40 min, and the absorbance was read at λ = 560 nm. The cells in each well were lysed in RIPA buffer, followed by total protein quantitation via the BCA assay, as a measurement of cell numbers for normalizing the urea results.

### 2.6. CYP 450 Assay

Coumarin (MilliporeSigma, St. Louis, MO, USA) was dissolved in DMSO (Fisher Scientific, Pittsburgh, PA, USA) at 100 mM. The stock solution was then serially diluted in phenol red free DMEM to a concentration of 100 µM, 250 µL of which was mixed with 250 µL of phenol red free DMEM and added to the cells for a final coumarin concentration of 50 µM. Cells were incubated for 45 min at 37 °C and then 100 µL of the media was taken for testing in a 96−well plate. Fluorescence intensity was measured using λ_ex_ = 410 nm and λ_em_ = 510 nm to observe the metabolic product of coumarin, 7−hydroxycoumarin. Signals were background corrected based on blank DMEM solutions and then normalized based on the number of cells as determined using the BCA assay.

### 2.7. MTS Assay (Proliferation)

A total of 250 µL of fresh phenol red free DMEM was added to each well along with 25 µL of MTS reagent (Abcam, Waltham, MA, USA). Cells were then incubated for 4 h at 37 °C. Next, 200 µL of each sample was transferred to a 96−well plate, and the absorbance was read at 490 nm. Signals were then normalized based on the number of cells as determined using the BCA assay.

### 2.8. Albumin Detection

After 24 h of culture, 10 µL of media was taken from each cell well and diluted in PBS by 100 times. A human albumin ELISA kit (Thermo Fisher Scientific, Pittsburgh, PA, USA) was used to quantitate the albumin production from the Huh7 cells cultured on the fibrous vs. flat substrates. Lysing the cells and quantitating the total protein via the BCA assay generated cell numbers in each well, which was used to normalize the albumin measurements.

### 2.9. Intracellular Amino Acid Quantitation Using LCMS (Liquid Chromatography Mass Spectrometry)

Cells on fiber/flat were lysed. Briefly, cells were washed with ice cold (0 °C) PBS three times followed by adding 400 µL LCMS lysing buffer (2/2/1, *v/v/v*, LCMS grade methanol/acetonitrile/H_2_O, MiliporeSigma, St. Louis, MO, USA) with 4 µM ^13^C−threonine (as the internal standard) added (Cambridge Isotope Laboratories, Inc., Tewksbury, MA, USA). Cell lysates were then sonicated with an ultrasonic homogenizer at 1 burst per 5 s and immediately incubated in dry ice. An aliquot of 200 µL of the lysate was used for the BCA assay. The remaining cell lysates were incubated for one hour at −20 °C to precipitate proteins, followed by centrifugation at 14,000 rpm (Eppendorf, Centrifuge 5424R, Enfield, CT, USA) at 4 °C for 15 min. After collecting the supernatant, the samples were lyophilized (Savant from Thermo Scientific, Waltham, MA, USA). The dried extracts were reconstituted in 15 µL LCMS grade water with 0.1% formic acid.

The Agilent capillary HPLC 1260 Infinity series coupled with G6530C LC−QTOF mass spectrometer was utilized for the amino acid analyses. Under positive mode, the mass scan range was 50–1000 *m/z*, mass accuracy 2 ppm, gas temperature 320 °C, drying gas 10 L/min, nebulizer 30 psi, capillary voltage 3500 V, fragmentor 110 V, skimmer 65 V, and OCT1 RF Vpp 750 V.

The sample inlet flow rate was 30 µL/min with a C18 Luna Omega column (1.6 µm, PS 100 Å with an inner diameter of 100 × 2.1 mm; Phenomenex, Torrance, CA, USA). Mobile phase A was 0.1% formic acid in LCMS grade water, and mobile phase B was LCMS grade methanol. The gradient started with 0% B and was increased to 100% B at 15 min. Then 100% B was held until 25 min and decreased to 0% at 30 min and remained at 0% B until 45 min. The injection volume was 5 µL by an autosampler.

### 2.10. Immunoblotting of Integrins

Cells on fiber/flat were washed with PBS and lysed by adding 100 µL RIPA buffer (radioimmunoprecipitation assay buffer with 150 mM sodium chloride, 1.0% NP−40, 0.5% sodium deoxycholate, 0.1% SDS and 50 mM Tris, pH 8.0) with complete protease inhibitor cocktail (Abcam, Waltham, MA, USA). Then, cell lysates were sonicated and immediately put on ice. The protein concentration was determined with the BCA assay. Lysates were added to the loading solution (2×) with 2−mercaptoethanol supplemented and heat−denatured at 95 °C. Then 25 µg of total protein was loaded to 8% SDS−polyacrylamide gels (BioRad, Hercules, CA, USA) followed by electrophoresis (110 V, 40 min). After electrophoresis, proteins were transferred into nitrocellulose membranes (BioRad, Hercules, CA, USA). Then, the membranes were incubated in PBST (PBS with 0.1% Tween−20) buffer with 2% non−fat dry milk powder at room temperature for 30 min. After being washed with PBST on a shaker for 3 times (10 min each), the membranes were cut into two pieces and incubated with rabbit−polyclonal anti−integrin β1 and rabbit−polyclonal anti−β actin (1:1000) antibodies (Thermo Fisher Scientific, Pittsburgh, PA, USA) at 4 °C overnight. Next, the membranes were washed 3 times again and then incubated with horseradish peroxidase−conjugated anti−rabbit antibodies (1:1000; Thermo Fisher Scientific, Pittsburgh, PA, USA). The Amersham Imager (GE Healthcare, Marlborough, MA, USA) was used for the detection of immunoreactivity. The band intensity was normalized to β actin in ImageJ.

### 2.11. mRNA Isolation and Quantitative mRNA Analyses

The flat and fibrous discs with cells therein were cut into small pieces and then transferred into Precellys lysing tubs with beads (Bertin corp, Rockville, MD, USA) in dry ice. Then, the cells on the substrates were homogenized with a Cryolys evolution tissue homogenizer (Bertin corp, Rockville, MD, USA) at 0 °C, followed by snap−freezing of the solution in dry ice. Total RNA in cells on fiber/flat were extracted with RNeasy Micro kit (QIAGEN, Germantown, MD, USA) following the manufacturer’s instructions. Then, the total RNA was quantified with the Biotek cytation 5 assay (BioTek, Winooski, VT, USA). A total of 0.5 µg RNA was aliquoted for reverse transcription (RT) analyses with the HighCapacity cDNA Reverse Transcription Kit (Applied Biosystems, San Francisco, CA, USA). Real−Time qPCR was then performed with the TaqMan™ Fast Advanced Master Mix (Applied Biosystems, San Francisco, CA, USA). The ITGB1 (Applied Biosystems, San Francisco, CA, USA) and internal control GAPDH (Applied Biosystems, San Francisco, CA, USA) primers were utilized with The LightCycler^®®^ 96 System (Roche, Indianapolis, IN, USA) for the analyses.

### 2.12. Inhibiting Integrin β1

The peptides RGDS (arginine−glycine−aspartic acid−serine, 50 µg/mL) and RGES (arginine−glycine−glutamic acid−serine, 50 µg/mL) as the negative control were added in the media separately with cells cultured on the flat surface for 48 h before metabolomics analysis.

### 2.13. Statistics

Replicate numbers of the experiments are shown in figure captions. Student’s t−tests were applied to compare differences. A significant difference was determined only when *p* < 0.05. For most figures, the errors represent the standard error of mean (S.E.M.) unless otherwise specified.

## 3. Results and Discussion

In vitro liver models are critical tools for drug development and pathophysiological studies [14]. It was recently acknowledged that hepatocytes cultured as a 2D monolayer cannot mimic the complex microenvironment of cells in vivo, and thus cause significant discrepancies between in vitro and in vivo results [21]. Although various studies have been reported showing that 3D scaffolding materials can enhance hepatocyte functions in vitro, few have investigated the impacts of the 3D cultures on the basic metabolism of the cells, nor the underneath mechanisms. The liver is essentially a metabolic organ in the body [22] and depicting the metabolic patterns will provide a broad perspective to understand the cell activities/functions. Therefore, we studied if and how a 3D scaffold could affect hepatic metabolism with a focus on the intracellular amino acids, which directly determine the protein synthesis of the liver, with new mechanistic insights discovered.

### 3.1. The Fibrous ECM and Basic Characterizations of the Cells

**Figure 1:**
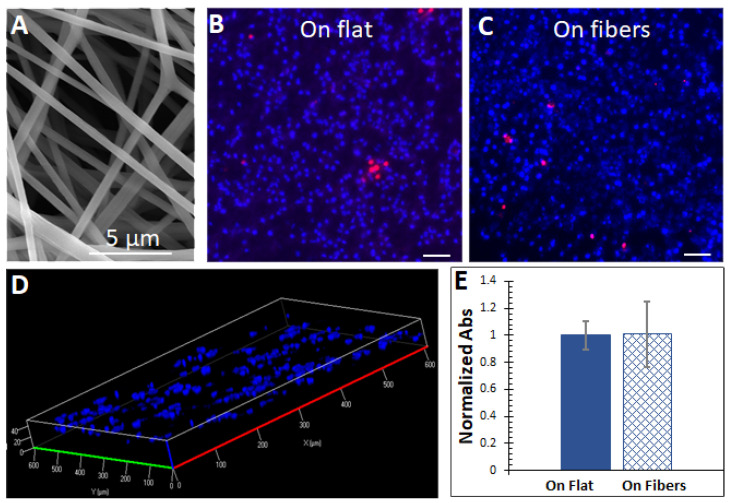
(**A**) A SEM view of the microfibers electrospun from silk fibroin. (**B**,**C**) Fluorescent imaging of cells on the flat surface and on the microfibers. Red = dead cells, blue = total cells. High viabilities were observed without significant differences on both substrates. Scale bars = 100 µm. (**D**) The cells could infiltrate the microfibrous scaffold through the inter−fiber pores up to 40 µm. (**E**) Cell proliferations on both substrates did not significantly differ. The MTS assay was used with absorbance (Abs) readings at 490 nm. The absorbance signals were normalized by cell numbers on each substrate. *n* = 5.

Silk fibroin is a protein derived from raw silk, which has been widely used for biomedical applications due to its superior properties, including low immunogenicity, similarity to native ECMs, and ease of production [11,23]. Previous works showed that electrospun fibers from silk fibroin with submicron diameters are suitable substrates for 3D cell cultures [11,24]. Therefore, as shown in Figure 1A, we applied silk fibroin fibers with an average diameter of 0.8 ± 0.2 µm (mean of 200 fibers from 5 samples ± standard deviation) as the ECM for hepatocytes, with the control being a flat 2D substrate. Although primary hepatocytes are available for research, due to the limited accessibility and high cost, hepatocyte cell lines derived from carcinoma are likely to be used widely for this purpose in the near future. Therefore, we used the well−characterized hepatic cell line Huh7 in this work. 

As shown in Figure 1B,C, the cells could grow and maintain viability on both the flat and fibrous scaffolds. Figure 1D shows that the cells could infiltrate through the microfibrous scaffold up to 40 µm. The proliferation of the cells was assessed by the MTS assay, which can be reduced by viable cells to a colored formazan dye. Absorbance signals read from the media of cells cultured on the substrates were normalized by cell numbers (lysing the cells and measuring the total protein amount) and did not differ significantly (Figure 1E).

Overall, these data suggested that the microfibrous ECM and the flat surface supported the cell culture without significant growth differences.

### 3.2. Characterization of the Basic Functions of the Liver Cells

**Figure 2:**
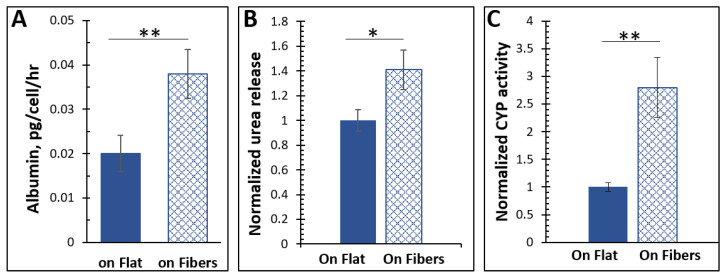
(**A**) The cells cultured on the microfibers produced significantly more albumin. *n* = 5, error = S.E.M., ** *p* < 0.025. (**B**) The cells on the fibers released more urea. *n* = 5, error = S.E.M., * *p* < 0.05. (**C**) The CYP450 activity in cells cultured on the microfibrous ECM was higher than those cultured on flat. *n* = 5, error = S.E.M., ** *p* < 0.025. All the measurements were conducted in the culturing media. The cell numbers of each sample were quantified by lysing the cells and measuring the total protein content, based on which the albumin, urea, and CYP450 results were normalized to per cell.

Hepatocytes constitutively synthesize albumin, the most abundant protein in blood plasma, accounting for 50% of the total protein in serum [25]. Albumin is critical for maintaining blood pH, transporting molecules throughout the body, sustaining oncotic pressure, and is necessary for various other roles as well [26,27]. As it is exclusively produced by the liver and is a key component for maintaining homeostasis, it is a common marker for a functioning liver or liver model. We, therefore, measured the albumin production of the liver cells on the fibers vs. on the flat surface using ELISA kits. As demonstrated in Figure 2A, after normalizing by cell number, the hepatocytes on the microfibers produced almost twice as much albumin as those in flat cultures (*p* < 0.025), showing that the microfibers enhanced albumin production from the cells.

The liver is critical in removing toxic species from the bloodstream. As it sends blood directly to the heart to be distributed throughout the body, this function is essential. Ammonia is a common molecule in the body found either through direct absorption via dietary metabolism or through amino acids in the body [28]. Hepatocytes can convert ammonia to urea via the urea cycle [29,30,31]. Therefore, urea secretion is usually measured to assess hepatocyte functions [32]. Using a colorimetric enzyme assay, the urea secretion of the cells cultured on the two ECMs was measured and compared. The hepatocytes cultured on the microfibers released significantly more (*p* < 0.05) urea than cells on the flat surface.

The liver is also responsible for metabolizing xenobiotics as they are introduced to the body. Metabolism occurs predominantly via the CYP450 superfamily of hemeproteins [33]. Although CYP450 enzymes are involved in pathways regarding vitamin metabolism, cholesterol production, and the oxidation of unsaturated fatty acids [34], most of those tested for retained functionality in hepatocytes are those concerned with xenobiotic metabolism. Hence, we added to the cell culture media coumarin, which can be metabolized by the CYP450 to a fluorescent product, and measured the fluorescence in the media after 24 h. We found that fluorescence, and by inference the CYP450 activity, was significantly higher (*p* < 0.025) in cells grown on the fibrous scaffold than those as a flat 2D monolayer.

This data first confirmed successful cultures of hepatocytes because the three fingerprint hepatic functions were all observed. Moreover, the microfibrous ECM significantly enhanced the hepatic functions.

### 3.3. The Effects of the Fibrous ECM on Intracellular Amino Acids

**Figure 3:**
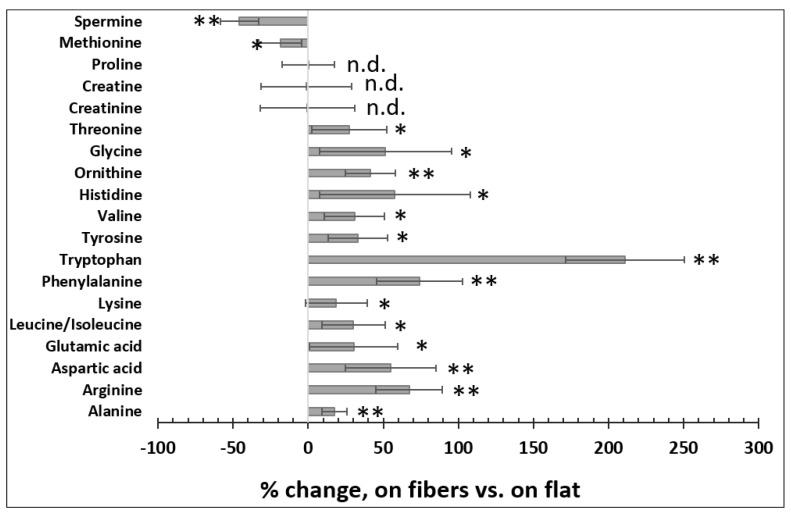
The microfibrous ECM changed intracellular amino acid levels. *n* = 10, error = S.E.M., * *p* < 0.05, ** *p* < 0.025, n.d. = no significant difference.

Amino acids play key roles in cells, among which about 20 species are not only essential building blocks for proteins [35], but are also linked to other metabolic pathways such as glycolysis and the TCA (tricarboxylic acid) cycle [36]. For example, glutamic acid and aspartic acid enter the TCA cycle after their conversion to TCA intermediates α−ketoglutarate and oxaloacetic acid, respectively [37]. The bioavailability of the essential amino acids is critical for cellular function, especially in hepatocytes, because the liver is an important organ for protein synthesis, degradation, detoxification, and amino acid metabolism [38,39]. Therefore, we measured the quantities of the 20 amino acids using LCMS in Huh7 cells cultured on the fibers vs. on flat. As shown in Figure 3, Huh7 cells cultured on the fibers tended to have higher levels of amino acids than those cultured on flat, particularly tryptophan, which was increased by >200%. On the other hand, cells cultured on the fibers had lower levels of spermine and methionine, but similar levels of proline, creatine, and creatinine compared to those cultured on the flat surface. Interestingly, indoleamine 2,3−dioxygenase 1, which degrades tryptophan and thus reduces tryptophan levels, is overproduced in human carcinoma hepatocyte cell lines including Huh7 compared to normal liver tissues [40,41]. This may explain, at least in part, why tryptophan levels were lower in Huh7 cells grown on the flat surface. The levels of the branched−chain amino acids (BCAAs), including leucine, isoleucine, and valine, were reduced in carcinoma hepatocytes [42]. Other studies also suggest that increasing intracellular BCAAs could suppress the growth rate of carcinoma hepatocytes [43,44]. Our results show that the levels of the BCAAs were increased in the cells cultured on the fibrous scaffold. Methionine metabolism is impaired in diseased hepatocytes, including hepatic carcinoma, which leads to elevated methionine levels in both hepatocytes and in serum [45,46,47]. Our data in Figure 3 also demonstrate that culturing cells on the fibers reduced the methionine level. Thus, at least for tryptophan, BCAAs, and methionine, the data indicated that the fibrous scaffold modulated the levels of these amino acids towards physiological relevance in Huh7 cells.

### 3.4. Mechanistic Studies of the ECM Effects

**Figure 4:**
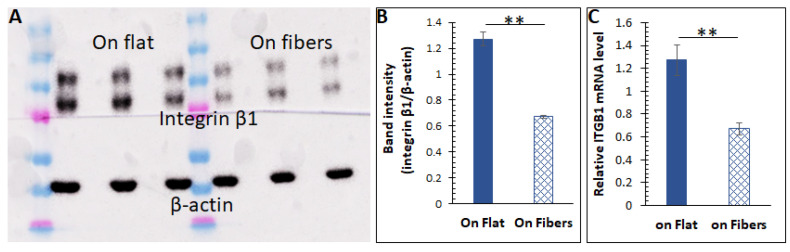
(**A**) Western blot results of integrin β1 from cells cultured on the flat surface vs. on the microfibers. The β−actin bands were also stained for intensity normalization. (**B**) Quantified band intensities for the Western blot. *n* = 5, error = stdev, ** *p* < 0.025. (**C**) Relative integrin β1 mRNA (ITGB1) levels in cells cultured on the two substrates. *n* = 10, error = S.E.M., ** *p* < 0.025.

In order to understand how the 3D scaffolding ECM modulates intracellular activities, we measured the expression of the integrins, a family of transmembrane ECM receptors. It is recognized recently that integrins are not just adhesion molecules localized to the cell–ECM contact areas but also affect intracellular signaling. For instance, integrin overexpression can suppress intracellular cAMP levels via the β−adrenergic receptor (β−AR) [48,49,50] and the G_α_ [51]. On a fibrous ECM, cells form pseudopodia structures to bind the fibers [52], where the integrins are expressed. However, on a flat stratum, one side of a cellular body is binding to the ECM, which can likely cause integrin overexpression compared to a fibrous substrate. As integrin β1 binds all ECM proteins [53] and has shown significant effects on cell behaviors/function in prior works by us and others, we hypothesized that the ECM microstructures varied the integrin expression, and thus changed the amino acid abundance. We first tested the hypothesis by measuring this integrin from cells cultured on the two substrates via Western blot. The results in Figure 4A,B suggested that cells cultured on the microfibers indeed had a lower amount of integrins. The intracellular ITGB1 (integrin β1) mRNA level was also lower in cells on the fibers (Figure 4C). All these data strongly indicate that the microfibrous ECM could decrease the expression of integrin β1.

To test the second part of the hypothesis (whether integrin overexpression caused the reduction in amino acid levels), we applied a comprehensive blocker (the RGDS peptide [54]) to partially inhibit integrin β1 on cells cultured on flat substrates, and re−analyzed the intracellular amino acids. Based on recent work by us [55], the proper dose of the inhibitor was determined to be 50 µg/mL. The peptide RGES with the third amino acid replaced by glutamic acid (E) was used as the negative control.

**Figure 5:**
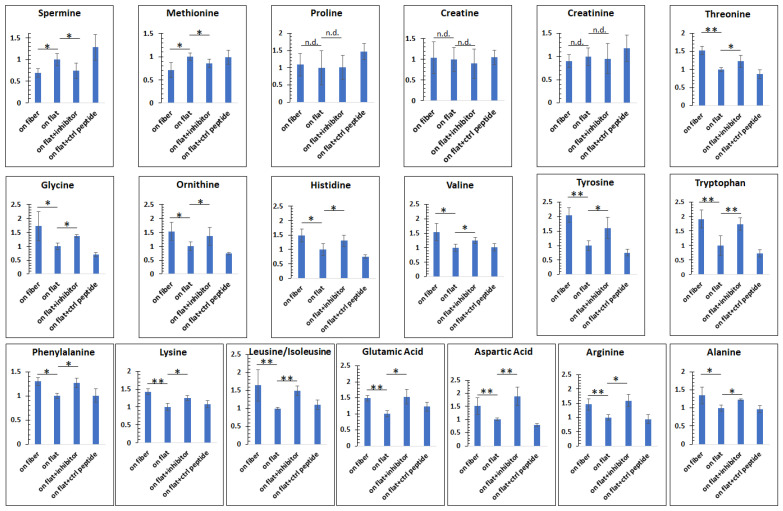
Re−analyses of the amino acids after blocking integrin β1. On fiber = cells cultured on the microfibers; on flat = cells cultured on the flat surface; on flat + inhibitor = cells were cultured on the flat substrate, but with 50 µg/mL RGDS (the inhibitor) in the media during culture; on flat + ctrl peptide = cells were cultured on the flat surface, but with 50 µg/mL RGES (the control peptide). After inhibiting integrin β1 on cells cultured on the flat ECM, the differences in amino acids levels from cells on the microfibers were minimized. The results suggest that integrin β1 was a mechanism transducing the ECM microstructural information to intracellular amino acid abundances. *n* ≥ 5, error = S.E.M., * *p* < 0.05, ** *p* < 0.025, n.d. = no significant difference.

As shown in Figure 5, we first reproduced the observations in Figure 3. For instance, there were less spermine and methionine in cells on the fibers, compared to those on flat; no significant differences were found for proline, creatine, and creatinine. The levels of the remaining amino acids were higher in cells on the fibers. However, with partial inhibition of the integrin β1 with the integrin inhibitor RGDS, the amino acid levels in cells cultured on the flat surface were elevated to a level close to those in cells cultured on the fibers. Taken together, these results show that hepatocytes respond to the microstructures of the ECM by altering integrin β1 expression, which, in turn, can regulate intracellular events/conditions such as amino acid production.

## 4. Conclusions

In this work, we focused on studying the effects of a commonly used 3D scaffold on intracellular amino acid levels in hepatocytes, which has not been studied heretofore. New knowledge that the fibrous ECM could increase the amount of most amino acids was generated. Although various studies have shown that 3D cell cultures can change cell functions, the mechanism was poorly understood. Our work here reduces this gap by showing that the ECM microstructures affect integrin expression, which alters intracellular signaling. The new knowledge enhances our understanding of ECM–cell interactions and will facilitate improved modeling of hepatocytes in the future.

## Data Availability

Not applicable.
